# Paroxysmal Atrial Fibrillation with Rapid Ventricular Response Following COVID-19 Nasopharyngeal Swab: A Case Report

**DOI:** 10.3390/reports6010015

**Published:** 2023-03-15

**Authors:** Michele Mattia Viscusi, Luca Ambrosio, Danilo Ricciardi, Fabio Mangiacapra, Annunziata Nusca, Luca Paolucci, Gian Paolo Ussia, Francesco Grigioni

**Affiliations:** 1 Unit of Cardiac Sciences, Department of Medicine, Università Campus Bio-Medico di Roma, Via Alvaro del Portillo 21, 00128 Roma, Italy; 2 Operative Research Unit of Orthopaedic and Trauma Surgery, Fondazione Policlinico Universitario Campus Bio-Medico, Via Alvaro del Portillo 200, 00128 Roma, Italy; 3 Research Unit of Orthopaedic and Trauma Surgery, Department of Medicine and Surgery, Università Campus Bio-Medico di Roma, Via Alvaro del Portillo 21, 00128 Roma, Italy

**Keywords:** COVID-19, SARS-CoV-2, atrial fibrillation, arrhythmia, nasopharyngeal swab, trigeminocardiac reflex

## Abstract

Nasopharyngeal (NP) swab sampling is a simple procedure that has become extremely popular in the coronavirus disease 2019 (COVID-19) era, with hundreds of million specimens collected every day. However, rare but serious complications have been reported following NP swab acquisition. Here we present a case of paroxysmal atrial fibrillation associated with NP specimen collection in a healthy healthcare provider undergoing COVID-19 testing during departmental screening. This response may have been caused by an exaggerated vagal tone triggered by the trigeminocardiac reflex. Less invasive collection methods, such as saliva testing, may be warranted in predisposed individuals.

## 1. Introduction

Atrial fibrillation (AF) is the most frequent arrhythmia in adults, with increased prevalence and incidence with advancing age [1]. AF is characterized by ectopic and chaotic electrical impulses suddenly triggering atrial contractions and consequently overriding the heart’s natural pacemaker, thus resulting in various symptoms, including irregular heart rate, palpitations, dizziness, shortness of breath, and tiredness [2]. Multiple precipitating pathophysiological mechanisms have been proposed (structural heart disease, hemodynamic overload, metabolic and/or genetic factors), and several of them are not fully understood. Additionally, some cases or AF may also result from a substantial perturbation within the critical factors of the cardiac autonomic nervous system, more specifically an extreme overdrive of either the sympathetic or parasympathetic nervous system. Interestingly, in hearts without any structural disease, vagal tone is predominant, thus explaining why vagal-mediated AF seems more relevant in healthy young adults with absence of detectable heart disease [3]. On the other hand, sympathetically-mediated AF is observed in the presence of underlying heart structural disease since its first effect is to induce vagal suppression [4]. Consistently, numerous studies have demonstrated that vagal-induced bradycardia may potentially lead to pro-arrhythmogenic effects on the atrial tissue, consisting of slowing of atrial conduction, shortening of the atrial effective refractory period and increasing dispersion of atrial refractoriness [3]. In this regard, this type of vagal nerve stimulation is commonly used to induce and maintain AF in several experimental models. In contrast, low-level of vagus nerve stimulation, not inducing bradycardia, has been shown to play anti-arrhythmogenic effects [5].

Nasopharyngeal (NP) swab collection has been widely employed to isolate viral and bacterial pathogens causing upper and lower respiratory tract infections in both outpatient and hospital settings [6]. Due to the severe acute respiratory syndrome coronavirus 2 (SARS-CoV-2) pandemic, the use of NP swabs has dramatically increased in order to early detect and reduce the spread of the infection throughout the world, with hundreds of million tests performed every day [7]. Furthermore, NP sampling is particularly frequent in certain subpopulations such as healthcare providers, who are often involved in departmental screening on a regular basis [8]. Although trivial, NP swab collection has been associated with rare but serious complications, including massive epistaxis, swab breakage requiring endoscopic removal, cerebrospinal fluid leak, and meningitis [9,10]. Moreover, recent reports have also documented the onset of syncope [11] and even asystole [12] following NP sampling, probably mediated by the trigeminocardiac reflex [13].

Here we describe a case of paroxysmal atrial fibrillation (PAF) with rapid ventricular response following NP swab testing for SARS-CoV-2 in a healthy young adult.

## 2. Case Report

A 27-year-old man (weight: 76 kg, height: 175 cm; body mass index: 24.81 kg/m^2^) presented in December 2020 at the Emergency Department of our institution (Fondazione Policlinico Universitario Campus Bio-Medico, Rome, Italy) with recurrence of PAF with rapid ventricular response following NP swab testing for SARS-CoV-2. In 2018 he reported the first and only previous arrhythmic episode as a consequence of occasional alcohol consumption with concomitant nausea and vomiting. He was a nonsmoker and additional risk factors such as gastroesophageal reflux disease, hypertension, diabetes, and hyperthyroidism were ruled out. He also denied any history of drug assumption.

**Figure 1:**
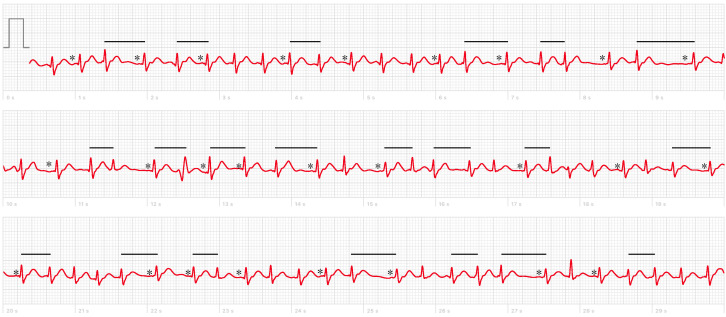
Baseline 1-lead ECG recorded by the patient using an Apple Watch Series 4 upon onset of palpitations showing narrow-complex tachycardia with rapid irregular ventricular rate (black lines) and absence of distinct repeating P waves (black asterisks).

Immediately after NP swab testing, he complained of rapid and irregular palpitations and recorded an electrocardiogram (ECG) using his Apple Watch (Series 4, Apple Inc., Cupertino, CA, USA), which was suggestive of atrial fibrillation (AF; Figure 1). Interestingly, the arrhythmic onset followed a transient sinus bradycardia occurring during NP sampling (Supplementary Figure S1). 

**Figure 2:**
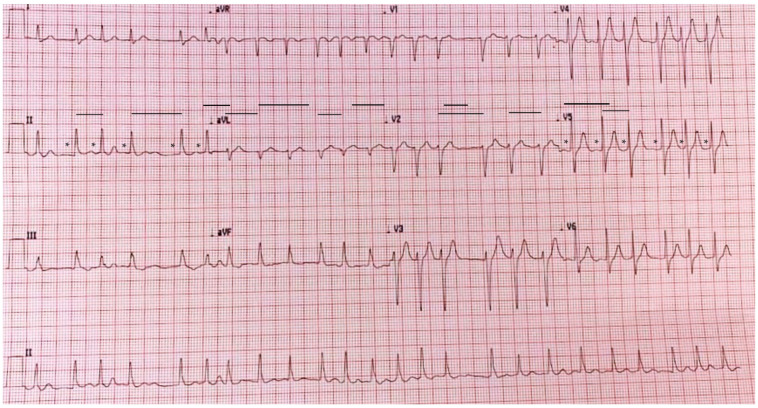
Standard 12-lead ECG showing narrow-complex tachycardia with rapid irregular ventricular rate (black lines) and absence of P waves (black asterisks).

A standard 12-lead ECG confirmed the diagnosis of AF with rapid ventricular response (150–170 bpm, Figure 2). The patient was subsequently hospitalized to attempt a rhythm control strategy and perform further investigations. 

On physical examination, his blood pressure was 140/80 mmHg, and peripheral oxygen saturation was 99% on room air. Calculated CHA_2_DS_2_-VASc and HAS-BLED scores were 0 and European Heart Rhythm Association (EHRA) scale was graded 2a.

Complete blood count, electrolytes, coagulation panel, inflammatory markers, liver and renal function, lipid profile, glycaemia, cardiac enzymes, and thyroid hormones were within the respective reference ranges (Table 1).

**Figure 3:**
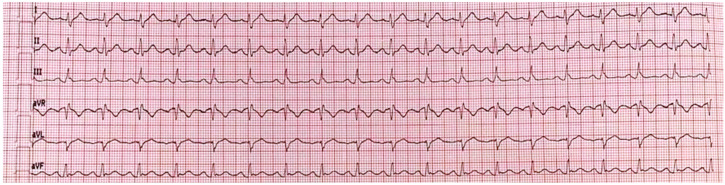
Peripheral-leads ECG showing conversion to regular sinus rhythm with normal ventricular rate.

Bedside transthoracic echocardiography quickly excluded valvular or other structural alterations and demonstrated normal left atrial (LA) volumes and left ventricular (LV) ejection fraction (65%). According to the last European Society of Cardiology (ESC) guidelines on AF [14], stroke risk was considered to be low (CHA_2_DS_2_-VASc = 0), and no anticoagulation was initiated. Since PAF episode duration was <48 h (approximately 3 h) and the patient was hemodynamically stable, pharmacological cardioversion with intravenous propafenone (2 mg/kg over 10 min) was initiated. Subsequently, stable sinus rhythm was restored and maintained throughout the whole hospitalization (Figure 3).

**Figure 4:**
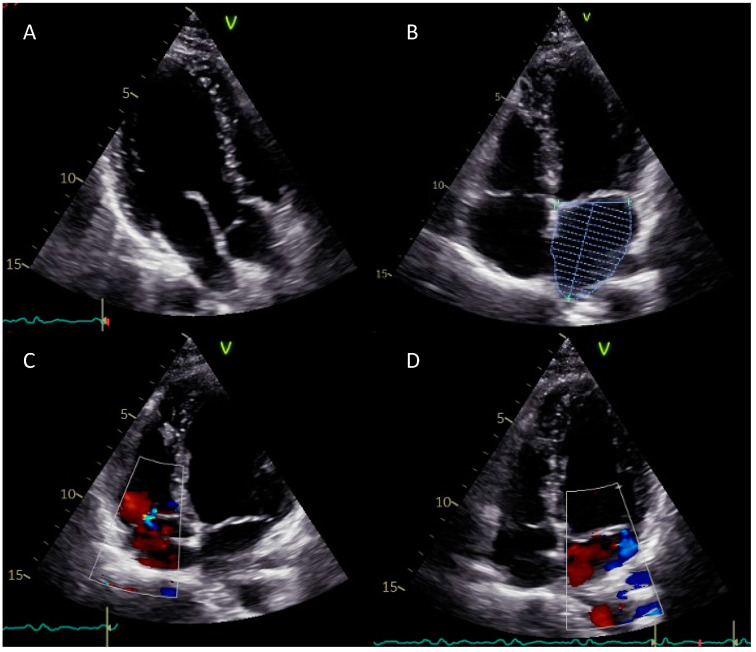
Complete baseline two-dimensional transthoracic echocardiography: three-chamber view (**A**); four-chamber view (**B**) with volumetric assessment of the left atrium (32 mL); color-Doppler four-chamber view (**C**,**D**). See text above for further details.

The following day, a transthoracic echocardiogram was performed (Figure 4). LV size and mass appeared to be normal with preserved global and regional systolic functions. Calculated ejection fraction was 62% with normal diastolic function. All visible segments of the ascending aorta and pulmonary artery were normal, as well as the atria and the right ventricle. The tricuspid valve showed a normal morphology with mild physiological regurgitation. The mitral valve showed minimal signs of anterior leaflet prolapse with mild associated regurgitation. Indirect systolic pulmonary pressure was within the physiologic ranges. Following the echocardiogram, the patient was discharged at home with optional post-cardioversion oral anticoagulation (60 mg edoxaban once daily for the following four weeks) considering the low stroke risk (CHA_2_DS_2_-VASc = 0). One month later, he underwent a cardiac magnetic resonance imaging (MRI) revealing no structural or functional abnormalities. Furthermore, he was advised to maintain a healthy lifestyle and to actively monitor any unusual subjective feeling that may anticipate an AF recurrence. The patient has been rigorously followed-up by our department for 1 year and showed or reported no recurrence of AF. He continued to participate in the COVID-19 departmental screening undergoing saliva polymerase chain reaction (PCR) testing.

## 3. Discussion

To our knowledge, this is the first case reporting an episode of PAF with rapid ventricular rate following NP swab testing for SARS-CoV-2 infection. The key messages of our case may be summarized as follows: (1) in patients with past medical history of PAF, a strict self-monitoring of cardiac rhythm is warranted in order to allow an early identification of potential arrhythmic recurrences [14]; (2) the so-called vagal-mediated atrial fibrillation, despite currently being an underdiagnosed phenotype of AF, appears to be predominantly prevalent in healthy young patients [3]; (3) although being the very first tool for early diagnosis of SARS-CoV-2 infection, NP swab collection may be associated with increased risk of complications within specific sub-group of populations such as patients with abnormalities of vagal tone triggering PAF.

Several novel methodologies of rhythm monitoring have been validated in the past few years and, among them, wearable devices with optical sensors able to detect irregular pulses reported the most robust evidence. In the Apple Heart Study, 34% of individuals who received a notification of arrhythmia (Apple Watch Series 1 through 3, Apple Inc., Cupertino, CA, USA) were later found to have AF and the positive predictive value in participants notified of an irregular pulse resulted 0.84 [15]. Consequently, the latest ESC Guidelines on Diagnosis and Management of AF recommended for a proper diagnosis of clinical AF a single-lead ECG tracing of ≥30 s showing heart rhythm with no discernible repeating P waves and irregular RR intervals, alternatively to a standard 12-lead ECG [9]. In this regard, our case was a clear example of symptom-driven cardiac rhythm self-monitoring, thus bearing to prompt AF diagnosis and treatment.

**Figure 5:**
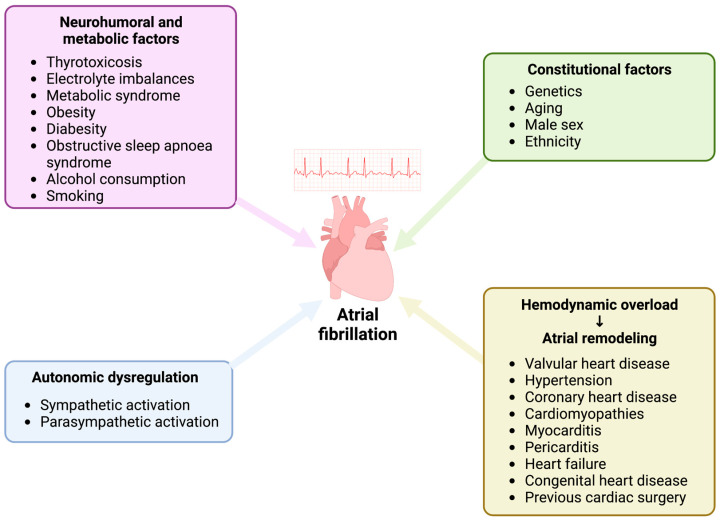
Potential pathophysiological mechanisms of AF. Created with www.biorender.com.

Despite the complex inter-play between all the potential mechanisms underlying its arrhythmogenesis (structural heart disease, hemodynamic overload, metabolic and/or genetic factors, Figure 5), in some cases AF may result from an imbalance within the key components of the cardiac autonomic nervous system, particularly an exaggerated stimulation of either sympathetic or parasympathetic nervous system. Indeed, increased sympathetic tone leads to focal ectopic firing and consequent AF via enhanced early afterdepolarizations (EADs) or delayed afterdepolarizations (DADs), whereas augmented vagal stimulation may trigger AF facilitating the development and maintenance of reentry mechanisms by shortening the atrial effective refractory period [4]. Interestingly, in hearts without any structural impairment or pathological remodeling, vagal tone is predominant, thus explaining that vagal-mediated PAF seems more relevant in young male adults with absence of detectable heart disease. On the other hand, sympathetically mediated AF is observed in the presence of underlying heart disease since its first effect is to induce vagal suppression [16].

In the past two years several cases reporting complications with NP swabs have been described: massive epistaxis, swab breakage requiring endoscopic removal, cerebrospinal fluid leak, and meningitis [4,5]. Moreover, recent reports have also documented the onset of syncope [6] and even asystole [7] following NP sampling, probably mediated by the well-known trigemino-cardiac reflex [8]. The latter is described as a brainstem reflex elicited by surgical manipulation or direct stimulation of any branch of the trigeminal nerve resulting in a vagal mediated-hemodynamic response leading to bradycardia (a drop of heart rate below of 60/min or 20% or more from the baseline and/or asystole) and/or hypotension (drop of systolic and diastolic arterial blood pressure below 90/60 mmHg or 70 mmHg of mean arterial blood pressure) [17]. The afferent limb of the reflex is constituted by the nerve endings of the sensory fibers of the V cranial nerve, which transmit through the Gasserian ganglion to the sensory nucleus of the trigeminal nerve, located on the floor of the fourth ventricle. Small internuncial fibers of the reticular formation connect the sensory nucleus of the trigeminal nerve with neurons located in the nucleus ambiguous and dorsal motor nucleus of the vagus nerve, whose cardioinhibitory parasympathetic fibers form the efferent limb [13]. In our case, the forceful stimulation of the deep nasal mucosa during NP sampling, innervated by sensory branches of the ophthalmic (V_1_) and maxillary (V_2_) nerves, has likely triggered the trigeminocardiac reflex leading to an exaggerated cardioinhibitory vagal response, thus resulting in PAF [3].

## 4. Conclusions

In this report, we described the case of a healthy young-male patient with a previous episode of PAF occurring after a considerable alcohol intake with nausea and vomiting and a present recurrence of arrhythmia following NP swab testing for SARS-CoV-2. Based on prior considerations, we may speculate that the patient is affected by vagal-induced PAF and therefore should avoid any potential triggering situation, including the execution of a traditional NP swab since it may induce an extreme response to the trigemino-cardiac reflex with consequent enhanced vagal stimulation and PAF development. Indeed, any alternative and less invasive methods of COVID-19 screening (i.e., saliva testing) are warranted in order to prevent any potential serious, albeit rare, complications in specific high-risk sub-group of population.

## Figures and Tables

**Table 1:** Laboratory values of the patient on admission.

Laboratory Parameter (Unit of Measure)	Value	Normal Range
Hb (g/dL)	15.50	13.50–17.50
RBC (×10^6^/μL)	5.09	4.30–5.50
WBC (×10^3^/μL)	6.70	4.00–10.00
HCT (%)	46.00	40.00–50.00
MCV (fl)	90.40	83.00–101.00
RDW (%)	12.40	11.00–16.00
PLT (×10^3^/μL)	196.00	150.00–400.00
AST (U/L)	38.00	5.00–34.00
ALT (U/L)	51.00	0.00–55.00
GGT (U/L)	14.00	11.00–59.00
CK (UI/L)	161.00	30.00–200.00
Plasma sodium (mmol/L)	140.00	136.00–145.00
Plasma potassium (mmol/L)	4.10	3.50–5.10
Creatinine (mg/dL)	0.84	0.73–1.18
Cholesterol (mg/dL)	211.00	<200.00 (optimal value)
HDL cholesterol (mg/dL)	52.00	>40 (optimal value)
LDL cholesterol (mg/dL)	138.00	<100.00 (optimal value)
Triglycerides (mg/dL)	151.00	0.00–149.00
Plasma glucose (mg/dL)	96.00	74.00–106.00
Blood urea (mg/dL)	34.00	19.00–43.00
Uric acid (mg/dL)	5.30	3.50–7.20
APTT (seconds)	31.40	23.00–32.00
INR	1.02	0.80–1.20
CRP (mg/dL)	0.05	<0.50
Troponin I (pg/mL)	<10.0	0.00–34.20
CKMB mass (ng/mL)	<0.3	0.00–5.20
Myoglobin (ng/mL)	38.00	0.00–154.90
TSH (μUI/mL)	1.96	0.35–4.94
fT4 (pmol/L)	11.10	9.01–19.50
fT3 (pmol/L)	5.76	2.89–4.88
COVID-19 E gene	Negative	-
COVID-19 N gene	Negative	-
COVID-19 RdRp/s gene	Negative	-

ALT = alanine transaminase; APTT = activated partial thromboplastin time; AST = aspartate transaminase; CK = creatine kinase; CKMB = creatine kinase isoenzyme MB; CRP = C reactive protein; GGT = gamma-glutamyltransferase; Hb = hemoglobin; HDL = high-density lipoprotein; INR = international normalized ratio; LDL = low-density lipoprotein; MCV = mean corpuscular volume; PLT = platelets; RBC = red blood cells; RDW = red blood cells distribution width; WBC = white blood cells.

## Data Availability

The data presented in this study are available on request from the corresponding author. The data are not publicly available due to privacy.

## Supplementary Materials

The following supporting information can be downloaded at: https://www.mdpi.com/article/10.3390/reports6010015/s1, Figure S1: Apple Health app data showing sinus bradycardia (left screenshot) followed by ECG recording (right screenshot).

## References

[1] Schnabel R.B., Yin X., Gona P., Larson M.G., Beiser A.S., McManus D.D., Newton-Cheh C., Lubitz S.A., Magnani J.W., Ellinor P.T. (2015). 50 year trends in atrial fibrillation prevalence, incidence, risk factors, and mortality in the Framingham Heart Study: A cohort study. Lancet.

[2] Brundel B.J.J.M., Ai X., Hills M.T., Kuipers M.F., Lip G.Y.H., de Groot N.M.S. (2022). Atrial fibrillation. Nat. Rev. Dis. Prim..

[3] Carpenter A., Frontera A., Bond R., Duncan E., Thomas G. (2015). Vagal atrial fibrillation: What is it and should we treat it?. Int. J. Cardiol..

[4] Qin M., Zeng C., Liu X. (2019). The cardiac autonomic nervous system: A target for modulation of atrial fibrillation. Clin. Cardiol..

[5] Zhang Y., Ilsar I., Sabbah H.N., Ben David T., Mazgalev T.N. (2009). Relationship between right cervical vagus nerve stimulation and atrial fibrillation inducibility: Therapeutic intensities do not increase arrhythmogenesis. Heart Rhythm..

[6] Gritzfeld J.F., Roberts P., Roche L., El Batrawy S., Gordon S.B. (2011). Comparison between nasopharyngeal swab and nasal wash, using culture and PCR, in the detection of potential respiratory pathogens. BMC Res. Notes.

[7] Hasell J., Mathieu E., Beltekian D., Macdonald B., Giattino C., Ortiz-Ospina E., Roser M., Ritchie H. (2020). A cross-country database of COVID-19 testing. Sci. Data.

[8] Piras A., Rizzo D., Longoni E., Turra N., Urru S., Saba P.P., Musumano L., Bussu F. (2020). Nasopharyngeal swab collection in the suspicion of Covid-19. Am. J. Otolaryngol..

[9] Fabbris C., Cestaro W., Menegaldo A., Spinato G., Frezza D., Vijendren A., Borsetto D., Boscolo-Rizzo P. (2021). Is oro/nasopharyngeal swab for SARS-CoV-2 detection a safe procedure? Complications observed among a case series of 4876 consecutive swabs. Am. J. Otolaryngol..

[10] Clark J.H., Pang S., Naclerio R.M., Kashima M. (2021). Complications of nasal SARS-CoV-2 testing: A review. J. Investig. Med..

[11] Bloom W.R., Bloom T.D. (2022). Neurally Mediated Syncope Triggered by COVID-19 Nasopharyngeal Swab Specimen Collection: A Case Report. Allergy Rhinol..

[12] Madanat L., Khalife A., Sims M. (2021). Asystole During Nasopharyngeal Swab: Is COVID-19 to Blame?. Cureus.

[13] Chowdhury T., Mendelowith D., Golanov E., Spiriev T., Arasho B., Sandu N., Sadr-Eshkevari P., Meuwly C., Schaller B., Trigemino-Cardiac Reflex Examination Group (2015). Trigeminocardiac reflex: The current clinical and physiological knowledge. J. Neurosurg. Anesthesiol..

[14] Hindricks G., Potpara T., Dagres N., Arbelo E., Bax J.J., Blomström-Lundqvist C., Boriani G., Castella M., Dan G.-A., Dilaveris P.E. (2021). 2020 ESC Guidelines for the diagnosis and management of atrial fibrillation developed in collaboration with the European Association for Cardio-Thoracic Surgery (EACTS). Eur. Heart J..

[15] Perez M.V., Mahaffey K.W., Hedlin H., Rumsfeld J.S., Garcia A., Ferris T., Balasubramanian V., Russo A.M., Rajmane A., Cheung L. (2019). Large-Scale Assessment of a Smartwatch to Identify Atrial Fibrillation. N. Engl. J. Med..

[16] Khan A.A., Lip G.Y.H., Shantsila A. (2019). Heart rate variability in atrial fibrillation: The balance between sympathetic and parasympathetic nervous system. Eur. J. Clin. Investig..

[17] Meuwly C., Chowdhury T., Sandu N., Golanov E., Erne P., Rosemann T., Schaller B. (2017). Definition and Diagnosis of the Trigeminocardiac Reflex: A Grounded Theory Approach for an Update. Front. Neurol..

