# Supplementary material for: Paroxysmal Atrial Fibrillation with Rapid Ventricular Response Following COVID-19 Nasopharyngeal Swab: A Case Report
Source: Reports (MDPI). 2023 Mar 15;6(1):15. doi: 10.3390/reports6010015 (PMC12225394; doi:10.3390/reports6010015)
Supplement: Supplementary file 1 [file reports-06-00015-s001.zip › reports-2241564-supplementary.pdf]

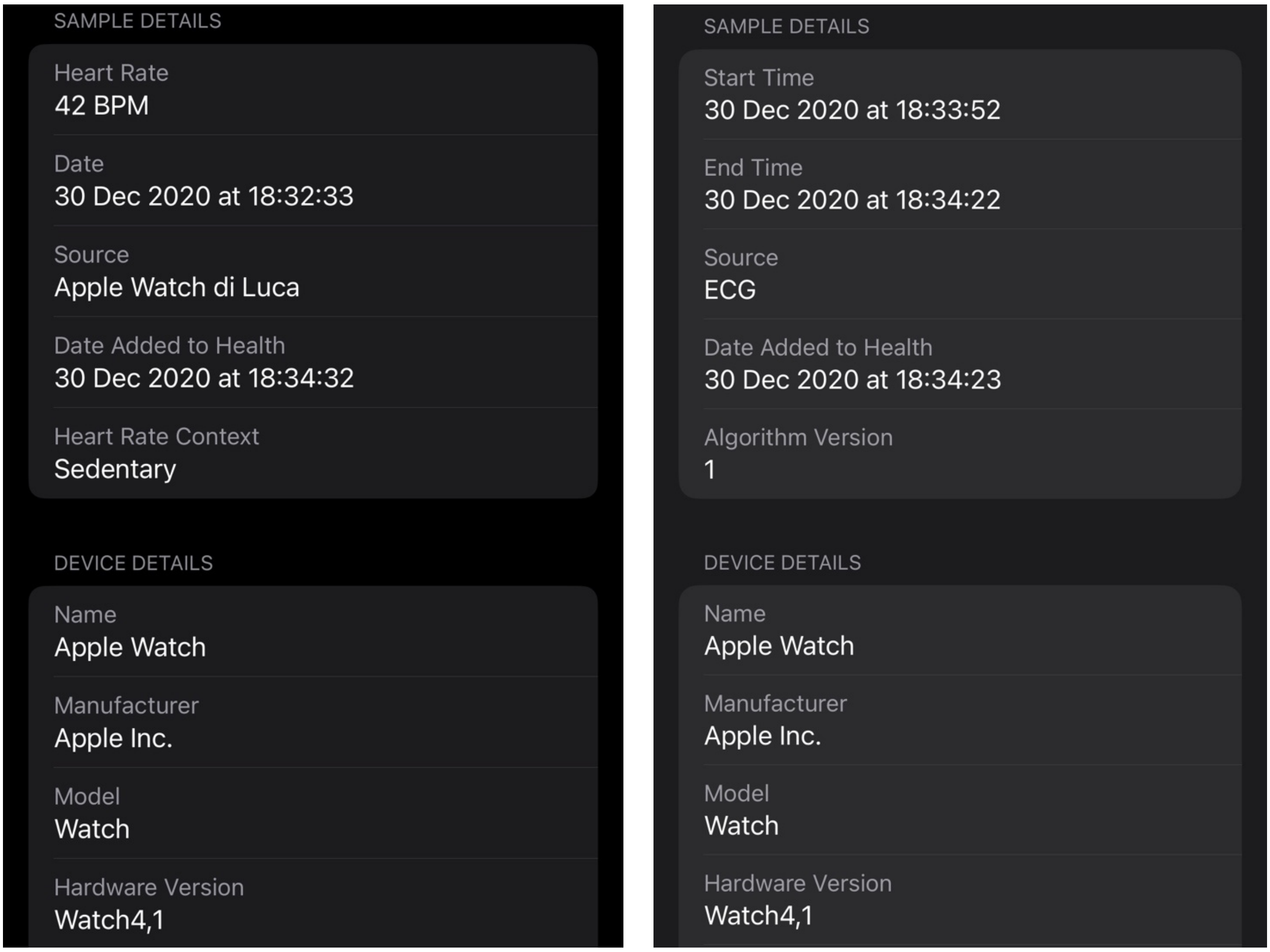

Figure S1: Apple Health app data showing sinus bradycardia (left screenshot) followed by ECG recording (right screenshot).
